# Circulating Tumor Cell Detection for Therapeutic and Prognostic Roles in Breast Cancer

**DOI:** 10.1002/cam4.70902

**Published:** 2025-05-28

**Authors:** Saiying Ma, Xiaojia Wang, Peter Ping Lin, Lei Lei

**Affiliations:** ^1^ Zhejiang Chinese Medical University Hangzhou China; ^2^ Zhejiang Cancer Hospital Hangzhou China; ^3^ Cytelligen San Diego California USA

**Keywords:** breast cancer, circulating tumor cell, circulating tumor endothelial cell, detecting technology, liquid biopsy

## Abstract

**Background:**

Circulating tumor cells (CTCs) are pivotal liquid biopsy (LB) biomarkers for breast cancer (BC), offering non‐invasive insights into tumor progression and metastasis. Despite their clinical promise, CTC detection remains technically challenging due to their extreme rarity in peripheral blood.

**Methods:**

This review systematically evaluates CTC detection methodologies, including immunoaffinity‐based approaches and biophysical techniques, which exhibit inherent trade‐offs in sensitivity, specificity, and compatibility with downstream analyses. Furthermore, post‐isolation molecular characterization methods spanning genomic, transcriptomic, and proteomic analyses are also critically assessed.

**Key Findings:**

CTC molecular profiling holds significant clinical relevance, enabling early diagnosis, prognostic stratification, and real‐time monitoring of therapeutic response. Baseline CTC counts or quantitative/phenotypic changes during treatment inform therapeutic decision‐making, predict drug resistance, and correlate with recurrence risk and metastatic progression.

**Conclusion:**

Multimodal analysis integrating CTC morphology, surface markers, and molecular alterations advances precision therapy. However, standardization of detection platforms and clinical validation of CTC‐guided protocols remain essential.

## Graphical abstract

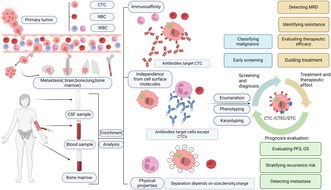

**Abbreviations**
ADC: antibody‐drug conjugate
ADH: atypical ductal hyperplasia
AR: androgen receptor
BBD: benign breast diseases
BC: breast cancer
CD31: pan‐leukocyte marker 31
CD45: pan‐leukocyte marker 45
CDK6: cyclin‐dependent kinase 6
CK: cytokeratin
CSFTCs: cerebrospinal fluid‐derived circulating tumor cells
CTC: circulating tumor cell
CTEC: circulating tumor endothelial cell
DAPI: 4′,6‐diamidino‐2‐phenylindole
DCIS: ductal carcinoma in situ
DFS: disease‐free survival
DNA: deoxyribonucleic acid
EBC: early breast cancer
EMT: epithelial‐mesenchymal transition
EpCAM: epithelial cell adhesion molecule
ER: estrogen receptor
EVs: extracellular vesicles
FDA: Food and Drug Administration
GO: graphene oxide
HB: herringbone
HER2: human epidermal growth factor receptor 2
IDC: invasive ductal carcinoma
iDFS: invasive disease‐free survival
LAHNSCC: locally advanced head and neck squamous cell carcinoma
LB: liquid biopsy
LM: leptomeningeal metastasis
LncRNAs: long non‐coding RNAs
MBC: metastatic breast cancer
MDR: multidrug resistance
MiRNAs: micro‐RNAs
MMPs: matrix metalloproteinases
MNPs: magnetic nanoprobes
MRD: minimal residual disease
MRI: magnetic resonance imaging
ncRNA: non‐coding RNA
OS: overall survival
pCR: pathologic complete response
PD‐L1: programmed death‐ligand 1
PFS: progression‐free survival
PSA: prostate‐specific antigen
RFS: recurrence‐free survival
RNA: ribonucleic acid
RNA‐ISH: RNA in situ hybridization
RT‐PCR: reverse transcription–polymerase chain reaction
SE‐iFISH: subtraction enrichment and immunostaining‐fluorescence in situ hybridization
SncRNAs: small non‐coding RNAs
TDEs: tumor‐derived exosomes
TDFs: tetrahedral DNA frameworks
TME: tumor microenvironment
Top1: topoisomerase 1
Trop‐2: trophoblast cell surface antigen‐2
TSGs: tumor suppressor genes
TTF: time to treatment failure
VEGF: vascular endothelial growth factor
VIM: vimentin

## 1 Introduction

Breast cancer (BC) is now the most common malignant tumor among women worldwide [1]. Despite advancements in clinical treatments leading to potential cures, BC remains the leading cause of cancer‐related mortality in women, with a notable proportion of patients experiencing recurrence and metastasis [2]. Early screening, appropriate treatment selection, and vigilant monitoring for metastatic recurrence are therefore imperative [3]. Although conventional imaging remains the cornerstone for tumor detection, its utility in early diagnosis is limited [4] and there are significant risks of over‐diagnosis and patient distress [5]. Histopathology, the gold standard for tumor staging, provides precise diagnostic insights [2] but is limited by its invasive nature and patient discomfort [5]. In contrast, liquid biopsy (LB), owing to its minimally invasive nature and ability for repeated sampling, has the potential to reveal the molecular landscape of neoplasms in real time, which could assist in monitoring the management of cancer during the treatment process [6].

One of the key biomarkers of LB is the circulating tumor cells (CTCs), the progenitors of metastatic tumors that circulate in the bloodstream either alone or in clusters [7]. However, CTCs exist at extremely low concentrations (few per milliliter of blood) owing to high attrition during metastasis [8]. Moreover, the process of epithelial‐mesenchymal transition (EMT) and the heterogeneity among CTCs pose challenges for their enrichment and detection [9]. To address these challenges, researchers are refining enrichment and detection technologies to increase sensitivity and specificity [10]. Beyond enumeration, CTCs carry noncoding RNAs (ncRNAs) and secrete exosomes, expanding their clinical utility as multifunctional biomarkers [11, 12].

CTCs serve as surrogate biomarkers for tumors [5]. Quantitative analysis (counting), phenotypic profiling, and karyotyping of CTCs enable therapy guidance, treatment response assessment, drug resistance identification, and prognostic stratification [13, 14]. Although CTCs are detectable across multiple cancer types, BC remains the primary focus of clinical CTC research [15], making it the central theme of this review.

## 2 Circulating Tumor Cells

The concept of CTCs was first described in 1869 by Ashworth, who identified cells exhibiting a tumor‐like morphology in the peripheral blood of a metastatic cancer patient [16]. Subsequent reports in the medical literature documented corroborated findings of tumor cells in the bloodstream. However, the higher incidence of CTC‐positive blood tests than of clinically evident distant metastases has raised concerns regarding their malignancy [9], prompting systematic investigations into the therapeutic and prognostic implications of CTCs [17].

In 1976, Nowel reconceptualized CTCs, suggesting that these cells derived from primary malignancies are capable of intravasating into the bloodstream or lymphatic system, thereby possessing the potential to metastasize to distant organs [18]. Subsequent studies further revealed the existence of CTC clusters [19], which, despite their low abundance in circulation, exhibit an enhanced capacity for dissemination and are associated with an inferior prognosis in affected patients [20]. The discovery of CTCs has stimulated significant scientific interest, driving innovations in CTC detection technologies and their application in oncology research.

## 3 Circulating Tumor Cell Detection

### 3.1 Positive Enrichment on the Basis of Immunoaffinity

Immunoaffinity methods are extensively utilized to capture cells through surface biomarkers, primarily using immunomagnetic separation with antibody‐coated magnetic particles. Positive enrichment targets tumor‐associated antigens [21], with epithelial cell adhesion molecule (EpCAM) being a preferred biomarker for identifying CTCs in epithelial‐originated malignancies. Captured CTCs are validated via standard criteria: 4′,6‐diamidino‐2‐phenylindole (DAPI)^+^/EpCAM^+^/cytokeratin (CK)^+^/the panleukocyte marker (CD45)^−^ [21, 22].

CellSearch, the first FDA‐authorized methodology for positive selection of CTCs [23], was initially developed in the late 1990s [24] and was approved for detecting metastatic breast cancer (MBC) patients in 2004 [25]. It combines anti‐EpCAM magnetic bead capture with fluorescent labeling for CK8/18/19, CD45, and DAPI, showing moderate sensitivity (60%–92%), high specificity (85%–98%) [26, 27], and substantial costs [28, 29, 30, 31]. In contrast, CytoSorter [32] employs analogous anti‐EpCAM‐based capture and immunofluorescence, achieving enhanced sensitivity (> 70%) in specific malignancies, such as locally advanced head and neck squamous cell carcinoma (LAHNSCC) [33, 34].

Instead of using EpCAM alone, the AdnaTest uses multiple tumor‐specific antibodies [35, 36, 37, 38]. After enrichment, CTCs undergo multiplex RT–PCR for tumor markers, with positivity requiring ≥ 1 marker above thresholds. Despite its sensitivity (60%–90%) being comparable to that of CellSearch [39], its cost‐effectiveness remains suboptimal [21, 40, 41].

MagSweeper [21, 42] processes whole blood without centrifugation or lysis, achieving high capture efficiency (70%–90%) and leukocyte‐free purity. Anti‐EpCAM magnetic beads bind CTCs, which are then collected via robotic magnetic rods at a speed that considers shear forces. Captured CTCs are microscopically validated and remain viable for downstream genomic analyses [43, 44].

Microfluidic systems improve capture by controlling flow dynamics. The CTC‐Chip, with anti‐EpCAM microcolumns, achieves 80%–95% sensitivity and 90%–97% specificity [45, 46, 47]. Postcapture enzymatic release facilitates CK/CD45‐based phenotyping [48]. Microcolumn arrays limit CTC detection and characterization because of their opacity, and subsequent microfluidic devices based on surface capture, such as herringbone (HB) and graphene oxide (GO) chips, have been developed to address these constraints [21]. T‐μFS is a highly efficient microfluidic system that combines tetrahedral DNA frameworks (TDFs), herringbone (HB) channel chips, and aptamer‐based reactions, achieving 80%–90% sensitivity and 85%–92% specificity for leukocyte‐free isolation [49]. Similarly, BioFluidica is a three‐modular system that employs antibody‐coated channels for multiphenotypic CTC capture, followed by trypsin release, impedance counting, and CD45/CK/vimentin (VIM) staining [50, 51, 52]. Despite incremental improvements in sensitivity and specificity, these technologies remain constrained by elevated operational costs and prolonged processing times [53].

Notably, the GILUPI CellCollector uniquely captures CTCs in vivo using an anti‐EpCAM‐coated wire inserted into veins, which can screen large volumes of blood [54, 55, 56, 57]. Postretrieval CTCs are stained with CD45/CK/DAPI for microscopy [58]. While superior sensitivity (90%–98%) is achieved, its specificity (85%–90%) and antibody‐associated expenses limit scalability [59].

### 3.2 Cell Surface Molecule‐Independent Enrichment

Negative enrichment isolates CTCs indirectly by removing background leukocytes using antigens not present on CTCs. While this approach avoids limitations of positive enrichment (e.g., capturing CTC subtypes lacking epithelial markers), it often reduces purity. However, some positive or negative devices can be interconverted by selecting different antibodies with high flexibility.

EasySep is an immunomagnetic technique based on negative enrichment [60]. The samples were incubated with antibodies such as CD45 conjugated to magnetic nanoparticles. Labeled blood cells are magnetically removed, leaving unbound cells (including CTCs) for EpCAM‐based immunofluorescence microscopy [61].

SE‐iFISH (subtraction enrichment and immunostaining‐fluorescence in situ hybridization) is a powerful tool for CTC analysis, allowing detailed assessment of cell characteristics (phenotype, chromosomal makeup, and morphology) with high detection accuracy [62, 63, 64]. It detects combinatorial abnormalities in protein biomarkers (e.g., CK, EpCAM, VIM, HER2, and PD‐L1) and chromosomal aneuploidy (e.g., chromosome 8 polysomy) tailored to tumor profiles [65, 66]. The process involves lysing red blood cells and removing leukocytes with magnetic beads coated with anti‐leukocyte antibodies. Precipitated cells were then smeared on slides, hybridized with chromosome 8‐specific FISH probes (Vysis CEP8), and labeled with fluorescent antibodies. Automated 3D microscopy identifies CTCs via tumor marker expression and chromosome 8 status (diploid/nondiploid) [62, 67, 68].

### 3.3 Enrichment on the Basis of Physical Properties

The reliance on EpCAM and CK as immunomagnetic targets poses significant limitations owing to their potential downregulation or subcellular relocalization during EMT. To address this, biomarker‐free methods isolate CTCs using physical differences between CTCs and hematopoietic cells—including morphology, deformability, and electric fields [42]—enabling label‐free capture better suited for downstream analysis. While cheaper than antibody‐based methods, these approaches often have lower efficiency and specificity [69].

Parsortix [70, 71, 72], approved by the FDA for MBC applications, captures CTCs in microfluidic chips based on cell size and flexibility [23, 73, 74]. Similarly, ClearCell is an automated cell recovery system cleared by the FDA that employs Dean Flow Fractionation (DFF) to separate larger CTCs from smaller blood cells for downstream assays [75, 76]. Vortex VTX‐1 [77] is another FDA‐approved technology that enriches CTCs using microscale fluid vortices that trap cells by size and shape [78].

Filtration‐based methodologies, such as the CanPatrol CTC system, employ membrane filters and vacuum pressure [79, 80, 81, 82]. After erythrocyte lysis, nucleated cells are size‐filtered, and retained CTCs undergo multiple RNA‐in situ hybridization (RNA‐ISH) coupled with immunofluorescence phenotyping [83]. Due to an inhomogeneous electric field, attractive or repulsive forces are exerted on the cells to separate them. ApoStream separates CTCs via electric fields. It guides CTCs to migrate toward high‐field regions for collection, whereas leukocytes are repelled via negative dielectrophoresis. Enriched CTCs are confirmed by microscopy [84, 85].

Emerging strategies also exploit the metabolic profile of malignant tumors. The Warburg effect—where cancer cells favor glucose breakdown via aerobic glycolysis, producing lactic acid—creates a negative cell surface charge [86]. Thus, magnetic nanoprobes (MNPs) have been engineered for charge‐selective CTC capture [87, 88, 89]. These fluorescent MNPs electrostatically bind to negatively charged cancer cells without prior labeling [90, 91]. The technologies mentioned above are summarized in Table 1.

**TABLE 1:** CTC detecting technologies mentioned.

Categorization	Technology	Description	Cancer type	Immunostaining	Whole blood	Sensitivity	Specificity	Limitation	Ref.
Immunoaffinity	CellSearch	Magnetic beads coated with anti‐EpCAM antibodies to enumerate epithelial CTCs	Breast, lung, prostate, colorectal cancer	CD45, CK, DAPI	NO	60%–92%	85%–98%	EpCAM dependency	[23, 26, 27, 28, 29, 30, 31]
	CytoSorter	Anti‐EpCAM antibody‐based capture of epithelial CTCs	Breast, lung cancer	EpCAM, CD45, DAPI	NO	> 70%	—	Technically complex workflow	[32, 33, 34]
	AdnaTest	Antibody‐coated beads combined with RT‐PCR for cancer‐specific CTC quantification	Breast, prostate, colorectal, ovarian cancer	Tumor‐specific markers (e.g., EpCAM, AR, AR‐V7)	YES	60%–90%	—	High sample stability requirements	[21, 35, 36, 37, 38, 39, 40, 41]
	MagSweeper	Robotic magnetic rods with antibody‐coated beads for CTC enumeration and genetic analysis	Breast, prostate cancer	EpCAM, CK, CD45, DAPI	YES	70%–90%	—	EpCAM dependency	[21, 42, 43, 44]
	CTC‐Chip	Microfluidic microcolumns with anti‐EpCAM antibodies for CTC isolation and analysis	Breast, lung, prostate, colorectal cancer	EpCAM, CK, CD4	YES	80%–95%	90%–97%	EpCAM dependency	[45, 46, 47, 48]
	T‐μFS	Microfluidic system integrating TDFs, HB‐chip, and apt‐HCR for live CTCs capture	Breast cancer	EpCAM, CK, MCF‐7	YES	80%–90%	85%–92%	Requires validation	[49]
	BioFluidica	Sinusoidal microsystem targeting FAP‐α and EpCAM for CTCs phenotyping	Breast, ovarian, pancreatic cancer	EpCAM, CD45, CK, VIM	YES	—	—	Technically complex workflow	[50, 51, 52]
	GILUPI CellCollector	In vivo antibody‐functionalized medical guidewire for EpCAM^+^ CTC capture	Breast, lung, prostate cancer	EpCAM, CD45, CK, DAPI	YES	90%–98%	85%–90%	Invasive procedure	[54, 55, 56, 57, 58, 59]
Surface molecule‐independent	EasySep	Anti‐CD45 magnetic nanoparticles for leukocyte depletion and unlabeled CTC enrichment	Breast cancer	CD45, EpCAM, CK	YES	—	—	Quantification only	[60, 61]
	SE‐iFISH	Subtractive enrichment combined with immunostaining and chromosomal analysis for viable CTCs identification	Breast, lung, gastric, pancreatic, ovarian cancer	EpCAM, VIM, HER2, PD‐L1, CK, DAPI, NCS	YES	85%–95%	90%–95%	Technically complex workflow	[62, 63, 64, 65, 66, 67, 68]
Physical properties	Parsortix	Microfluidic size/deformability‐based enrichment of unlabeled CTCs	Breast, lung, prostate cancer	EpCAM, CK, VIM	YES	—	—	Cell size bias	[23, 70, 71, 72, 73, 74]
	ClearCell	Spiral microfluidics system utilizing the Dean Flow Fractionation for size‐based CTC separation	Breast cancer, hepatocellular carcinoma	EpCAM, CK, VIM	YES	—	—	Flow rate restrictions	[75, 76]
	Vortex VTX‐1	Inertial microfluidic chip employing laminar microscale vortices for CTC isolation based on biophysical properties	Breast, lung cancer	CK, CD45, PD‐L1	YES	—	—	Potential cellular damage	[77, 78]
	CanPatrol	Filtration‐based system (vacuum‐driven) for unlabeled CTCs enrichment	Breast, pancreatic, lung cancer, hepatocellular carcinoma	EpCAM, CK, VIM, TWIST	YES	—	—	Background contamination	[79, 80, 81, 82, 83]
	Apostream	Dielectrophoretic field‐flow fractionation for leucocyte repulsion and unlabeled CTC enrichment	Breast, lung cancer	CK, EpCAM, E‐cadherin, VIM, β‐catenin, FRα	YES	—	—	Low sensitivity for rare CTCs.	[84, 85]
	Magnetic Nanoprobe (MNP)	Charge‐based magnetic nanoprobes for negatively charged CTCs enrichment	Lung, breast cancer	—	NO	—	—	Background contamination	[87, 88, 89, 90, 91]

**FIGURE 1:**
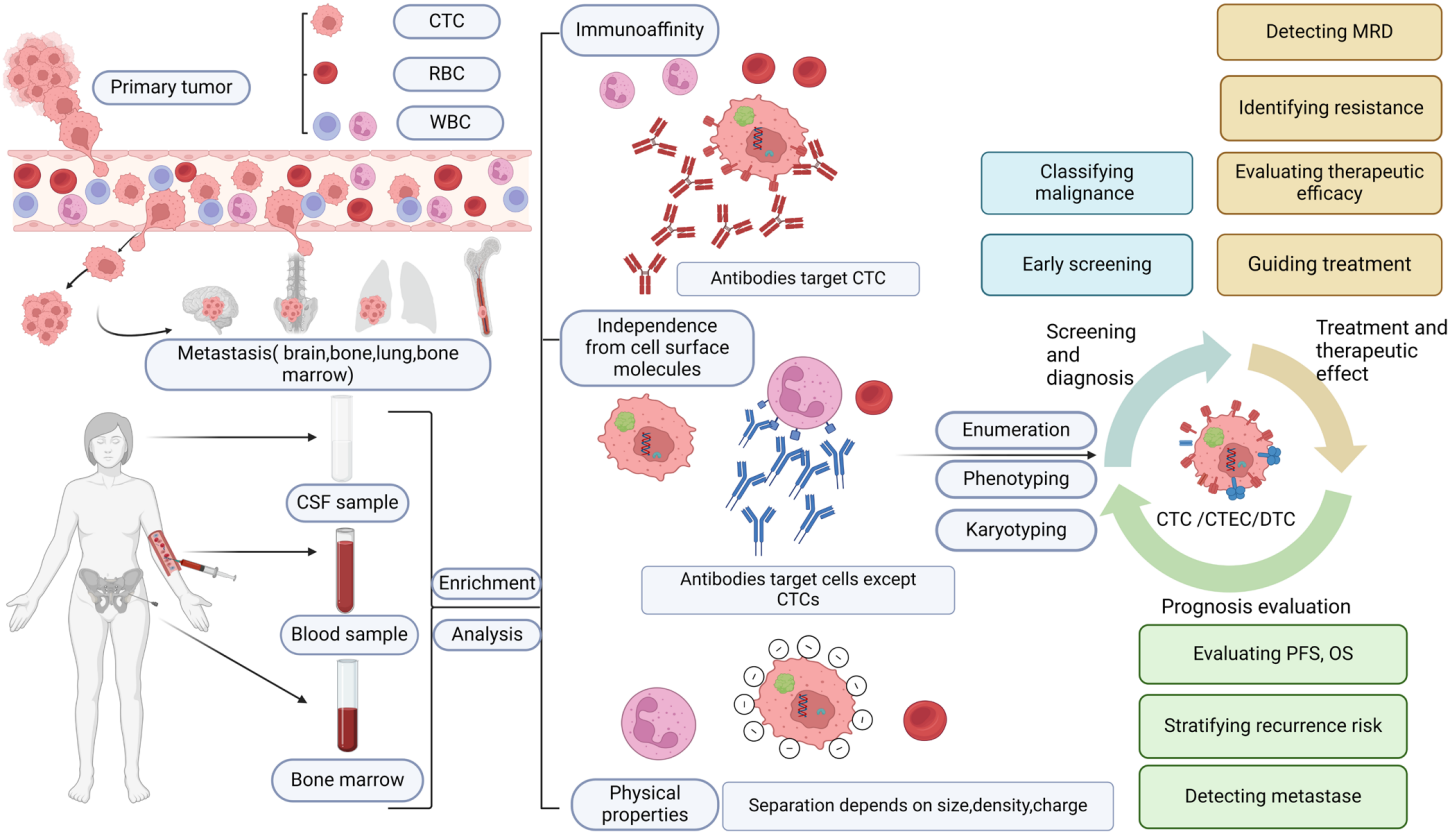
The figure outlines the process of tumor cell migration, the samples available for testing and the classification of enrichment assays, and the use of circulating cells. CSF: cerebrospinal fluid‐derived circulating tumor cell, CTC: circulating tumor cell, CTEC: circulating tumor endothelial cell, DTC: disseminated tumor cell, MRD: minimal residual disease, OS: overall survival, PFS: progression‐free survival, RBC: red blood cell, WBC: white blood cell/leucocyte. Source: Authors, via https://BioRender.com.

## 4 Clinical Applications of CTCs in Breast Cancer

### 4.1 Precancer Screening and Diagnosis

CTCs demonstrate emerging potential as biomarkers for early BC detection, with studies suggesting their capacity to identify malignancies prior to radiographic visualization [42]. Comparative analyses indicate that the yields of CTC‐based assays are comparable to those of conventional imaging modalities (e.g., ultrasonography, mammography, and MRI). Shao et al. [32] utilized the CytoSorter platform to detect CTCs. The study included 102 treatment‐naive BC patients, 177 patients with benign breast disease (BBD), and 64 healthy female patients. The detection rates were 91.2%, 40.7%, and 17.2%, respectively. Notably, when two CTCs were used as the cutoff value for stage I–III BC, the detection rates were 92.9%, 87.2%, and 100%, respectively, underscoring stage‐dependent utility [32]. However, CTCs remain excluded from clinical screening guidelines, in which the use of serum prostate‐specific antigen (PSA) to screen for prostate cancer is currently the only blood‐based screening biomarker recommended [92]. This disparity reflects persistent limitations in the clinical translation of CTCs, including low baseline detection rates. For example, a CellSearch‐based study reported detectable CTCs (≥ 1 CTC/7.5 mL) in only approximately 20% of confirmed BC patients, although detection rates positively correlated with tumor size, histologic grade, and lymph node involvement [93]. This underscores the insufficiency of CTC quantification as a standalone diagnostic tool, necessitating further validation through multicenter trials and standardized protocols [94] (Table 2).

**TABLE 2:** Some ongoing trials related to the clinical value of CTCs in breast cancer (according: https://clinicaltrials.gov; assessment update time: 12‐Feb‐2025).

Clinicaltrials.gov identifier	Title	No of patients	Time period	Primary endpoints
CTC as pre‐cancer screening and diagnosis				
NCT01322750	Circulating Tumor Cells (CTCs): A Potential Screening Test for Clinically Undetectable Breast Carcinoma	3125	Dec 2010–Jan 2023	CTCs detection
NCT02450357	The Detection of Circulating Tumor Cells (CTCs) in Patients With Breast Cancer Undergoing Cryosurgery Combined With DC‐CIK Treatment	60	Jun 2013–Dec 2015	CTCs detection
NCT03511859	Detecting Circulating Tumor Cells (CTCs) and Cell Free DNA (cfDNA) in Peripheral Blood of Breast Cancer (BC) Patients to Develop the Clinical Application for Early Detection and Diagnostics	210	Nov 2017–Dec 2019	CTCs detection
NCT03427450	Harvest of CTCs From MBC Patients Using the Parsortix PC1 System (HOMING)	421	Mar 2018–Dec 2019	Incidence of CTCs
NCT03958812	Diagnostic Power Comparison Between VOCs and CTCs	200	Jun 2019–Dec 2020	CTCs detection
NCT05633680	Circulating Tumor Cells Screen for Breast Cancer (CTCSFBC)	200	Sep 2019–Aug 2023	CTCs detection
NCT04239105	Detection of Circulating Tumor Cells in Breast Cancer Patients Using a Novel Microfluidic and Raman Spectrum Device	120	Jan 2020–Dec 2022	CTCs detection
NCT04241237	Concordance Between Liquid and Tissue Biopsy	120	Jul 2020–Dec 2022	CTCs detection
NCT04962529	Breast Cancer Liquid Biopsy Trial	450	Sep 2020–Jul 2023	CTCs detection
NCT06043661	Early Detection of Breast Cancer	700	Feb 2023–Dec 2025	CTCs detection
CTC for treatment guidance and efficacy assessment				
NCT01349842	Circulating Tumor Cells to Guide Chemotherapy for Metastatic Breast Cancer (CirCé01)	265	Mar 2010–Nov 2018	OS
NCT01322893	Enumeration and Molecular Characterization of Circulating Tumor Cells in Women With Metastatic Breast Cancer (CTC‐MBC)	150	Mar 2011–Jun 2016	CTCs detection
NCT01710605	Medico‐Economic Interest of Taking Into Account Circulating Tumor Cells (CTC) to Determine the Kind of First Line Treatment for Metastatic, Hormone‐Receptors Positive, Breast Cancers	800	Feb 2012–Sep 2018	PFS
NCT01619111	DETECT III—A Multicenter, Phase III Study to Compare Standard Therapy ± Lapatinib in HER2−ve MBC‐Patients With HER2+ve CTCs (DETECT III)	105	Feb 2012–Jan 2022	CTCs clearance rate
NCT01548677	Efficacy Study of Herceptin to Treat HER2‐Negative CTC Breast Cancer (TREAT‐CTC)	1317	Apr 2013–Mar 2017	CTCs detection
NCT04817501	Phenotypic Spectrum of CTCs in Tumors of the Female Reproductive System (CTCs)	150	Feb 2014–Dec 2022	CTC phenotype
NCT02123862	Cultured Circulating Tumor Cells in Prostate and Other Cancers	220	Apr 2014–Apr 2018	CTCs detection
NCT02449837	Investigation of Circulating Tumor Cells From Cancer Patients Undergoing Radiation Therapy	162	May 2014–Feb 2022	CTCs levels
NCT02602938	Aspirin on CTCs of Advanced Breast and Colorectal Cancer (ACABC)	40	Nov 2015–Feb 2017	CTCs detection
NCT03732339	CTC in Predicting Neoadjuvant Chemotherapy Among LABC Patients: a Single‐Center, Prospective, Exploratory Clinical Trial (CTCNeoBC)	29	Aug 2018–Jun 2019	CTCs detection
NCT03709134	Genomic Markers for Measuring Breast Cancer Response to Neoadjuvant Chemotherapy	100	Oct 2019–Sep 2022	pCR
NCT04059003	CTC Changes and Efficacy of Neoadjuvant Chemotherapy for Triple‐Negative Breast Cancer	200	Nov 2019–Aug 2024	CTCs detection
NCT03928210	Digoxin Induced Dissolution of CTC Clusters	58	Jul 2020–Dec 2023	CTCs detection
NCT04902937	Association of Adjuvant Radiotherapy of Non‐Metastatic Breast Carcinoma With Immunomodulation and Circulating Tumor Cell Phenotype in Relation to Patient Age (CETC)	200	Jul 2021–Dec 2026	CTEC counts
NCT05662345	ACT‐MBC: A Prospective Observational Impact Study of Circulating Tumor Cells (CTCs) in Metastatic Breast Cancer	65	Dec 2022–Aug 2026	CTCs detection
NCT04504747	Real Time Molecular Analysis of Breast Cancer Receiving Neo‐Adjuvant Chemotherapy (NEO‐R)	150	Nov 2022–Jan 2030	CTCs detection
NCT05834699	HER2 Expression of CTC to Predict Response in HER2‐Low Advanced Breast Cancer Patients Treated With ADC	50	Jan 2023–Apr 2025	PFS
NCT06067503	Biomarkers to Detect Endocrine Therapy Resistance	8	May 2024–Jan 2026	CTCs estrogen signaling
NCT06807502	Evaluation of Circulating Tumor Cells (CTC) Relevance in Breast Cancer Follow‐Up Using the ScreenCell Device (PROBE‐CTC)	93	Feb 2025–Nov 2026	CTCs detection
CTCs for prognostic monitor				
NCT02904161	Detection of Circulating Tumor Cells in Peripheral Blood From Healthy Volunteers and Patients With Cancer	137	Aug 2012–Dec 2016	OS
NCT02904135	Collection of Circulating Tumor Cells From the Peripheral Blood of Metastatic Breast Cancer Patients	141	Feb 2014–Dec 2016	OS
NCT05326295	Evaluation of Treatment Efficacy by Circulating Tumor Cell Phenotype Surveillance in Breast Cancer Patients	1000	Mar 2019–Mar 2029	iDFS
NCT04065321	Circulating Tumor Cell Detection in Patients With Luminal A Breast Cancer	500	Oct 2019–Sep 2029	DFS
NCT04818125	Circulating Cancer Cells/Macrophage HYbrid Cells in Patients With Breast Cancer. (CARMMYC)	61	Mar 2021–Jul 2022	Rate of CTCs
NCT04993014	Circulating Tumor Cells and Treatment De‐Escalation After Neoadjuvant Therapy for HER2 Positive Breast Cancer (HER2 Cell)	80	Mar 2021–Apr 2028	DFS
NCT05360290	CTCs in Breast Cancer After Neoadjuvant Treatment and Surgery: A Multicenter, Prospective Clinical Trial (CTCNeoBC‐E)	484	Sep 2022–Jun 2029	iDFS
NCT06048835	Circulating Tumor Cells Characterization in Breast Cancer Patients (BioCellPhe)	80	Dec 2022–Dec 2024	CTC detection
NCT05834686	HER2 Expression of CTC to Predict Response in HER2‐positive Advanced Breast Cancer Patients Treated With ADC	50	Jan 2023–Apr 2025	PFS

Abbreviations: DFS: disease‐free survival, iDFS: invasive disease‐free survival, OS: overall survival, pCR: pathologic complete response, PFS: progression‐free survival.

### 4.2 Treatment Guidance and Efficacy Assessment

The molecular profiling of CTCs offers a transformative approach to precision oncology in BC, enabling real‐time therapeutic guidance through biomarker‐driven strategies. Androgen receptor (AR) expression on CTCs indicates the efficacy of AR inhibitors [95], whereas estrogen receptor (ER) status indicates the effectiveness of ER‐targeted therapies [96]. Similarly, the detection of topoisomerase 1 (Top1^+^) CTCs can identify patients who are likely to derive overall survival (OS) benefits from Top1 inhibitors [97]. PD‐L1 detection provides a valuable predictive and prognostic biomarker for anti‐PD‐1 immunotherapy responsiveness [92], and patients with PD‐1^+^ CTCs benefit from anti‐PD‐1 immunotherapy. Zhou et al. [98] classified 26 patients into PD‐L1‐negative, PD‐L1‐low, PD‐L1‐intermediate, and PD‐L1‐high groups on the basis of CTC PD‐L1 expression, revealing dynamic biomarker fluctuations and superior clinical outcomes (PFS/OS) in high‐expression cohorts receiving immunotherapy. In hormone receptor‐positive MBC, CTC enumeration serves as a critical determinant for therapeutic stratification. A STIC clinical trial [99, 100] using CellSearch randomized hormone receptor‐positive, HER2‐negative advanced patients into CTC‐guided arms (≥ 5 CTCs/7.5 mL: chemotherapy; < 5 CTCs/7.5 mL: endocrine therapy) versus clinician‐choice arms. CTC‐guided management yielded superior median PFS (15.5 vs. 13.9 months) and OS (51.3 vs. 45.5 months), underscoring the prognostic utility of CTCs in optimizing treatment algorithms. Adjuvant radiotherapy confers recurrence‐free survival (RFS) and OS benefits in CTC‐positive patients, highlighting the role of CTCs in residual risk stratification [101]. Notably, HER2^+^ CTCs can be present in the peripheral blood of patients whose primary tumor is HER2^−^, validating the efficacy of HER2‐targeted therapy in this subset. A phase III clinical trial demonstrated that lapatinib administration in HER2^−^ MBC patients with HER2^+^ CTCs induced rapid CTC clearance, which was correlated with improved PFS and OS [94], thereby supporting the use of CTC‐based HER2 phenotyping as a therapeutic imperative.

Longitudinal CTC enumeration and phenotypic profiling allow for earlier identification of disease progression and therapeutic resistance [102]. CTCs were detected with CanPatrol, which revealed that treatment responders presented reduced total CTC counts or a predominance of epithelial CTCs, whereas disease progression was associated with elevated total CTCs or the proportion of mesenchymal CTCs [83]. Furthermore, SE‐iFISH identifies CD31^+^ CTECs, which are generated by the endothelialization of tumor cells or the cancerization of endothelial cells and are thought to be associated with angiogenesis. CTECs and CTCs constitute a unique pair of cellular circulating tumor markers [103]. During neoadjuvant therapy, CTECs demonstrate a biphasic response—an initial surge followed by a posttreatment decline [104]. High expression of Vim may increase the possibility of transendothelial migration of tumor cells and their transformation into CTCs; therefore, CTEC/CTC coamplification results in an increased likelihood of drug resistance, early recurrence of metastases, and poor DFS [41].

SE‐iFISH further extends to cerebrospinal fluid‐derived CTCs (CSFTCs) in leptomeningeal metastasis (LM)—a lethal complication of BC [105]—to monitor tumor progression and response to treatment and determine the number of treatments. The upregulation of CK18 is associated with progression, cell migration, metastasis, and recurrence. Intrathecal chemotherapy can reduce the burden of CSFTCs and CK18. However, < 6 cycles provoke rebound proliferation, necessitating sustained treatment [106]. Exome sequencing of CSFTCs revealed potentially actionable mutations, such as CDK6 V77G mutations, with the CDK4/6 inhibitor palbociclib demonstrating preclinical efficacy, suggesting that analyzing CSFTC moleculars can identify mutant drug targets and assist in personalized drug susceptibility testing [105].

A considerable number of early breast cancers (EBCs) recur 5–20 years after treatment, suggesting that minimal residual disease (MRD) may be present. CTCs exhibit unparalleled sensitivity in detecting MRD, identifying subclinical metastases ≥ 4 years prior to being clinically detected [107]. This capacity positions CTCs as a cornerstone for post‐treatment surveillance in EBC.

### 4.3 Prognostic Monitoring

The presence of ≥ 1 CTC/7.5 mL at baseline in EBC correlates with early metastatic recurrence and poor prognosis [93, 108]. Bidard and colleagues [109] demonstrated that detectable CTCs predict larger tumors, inferior survival outcomes, elevated risk of early recurrence, and reduced pathological complete response (pCR). However, CTCs and pCR exhibit minimal correlation, functioning as independent prognostic factors [94, 109]. Perioperative CTC and CTEC enumeration further refine the prognosis. A preoperative CTEC count ≥ 2 independently predicts decreased PFS in BC patients [110], whereas postoperative CTC counts strongly correlate with PFS. Postradiotherapy CTC escalation is associated with reduced DFS and a heightened risk of recurring metastases [101]. Adjuvant therapy aims to eradicate residual micrometastases, with pre‐ and postadjuvant chemotherapy CTCs serving as independent predictors of poor DFS and OS. Longitudinal CTC surveillance identifies patients at elevated risk for late recurrence [111], as evidenced by a study of 574 patients in which CTCs were detected in 96 (2‐year follow‐up) and 47 (5‐year follow‐up) cases [112]. Two‐year CTC detection (median one‐cell count) confers a 3.9‐fold increased mortality risk and 2.3‐fold increased relapse risk [113]. Five‐year CTC positivity is associated with a 6‐fold increased risk of recurrence [111].

For MBC, CTC counts ≥ 5/7.5 mL stratify patients into distinct prognostic cohorts [93]. Galardi et al. [96] stratified MBC patients on the basis of serial CTC monitoring and discovered that ≥ 1 CTC after the first treatment cycle predicted inferior PFS compared with their CTC‐negative counterparts. A ≥ 3 CTC increase from baseline was associated with a shorter PFS, whereas a posttreatment ≥ 5 CTC increase was associated with a shorter time to treatment failure (TTF). For hormone receptor‐positive/HER2‐negative MBC, the CTC‐driven classification (“Stage IV‐aggressive”: ≥ 5 CTCs; “Stage IV‐indolent”: < 5 CTCs) independently predicts OS, with the indolent subgroup deriving maximal PFS/OS benefits from CDK4/6 inhibitors (e.g., abemaciclib) [114].

CTC–CTEC interactions synergistically drive lymphatic and hematogenous dissemination, while phenotypic profiling reveals the metastatic potential of these interactions. The immunological checkpoint PD‐L1 and the mesenchymal‐type marker Vim expressed on CTCs/CTECs indicate increased invasiveness and poor prognosis in MBC. Vardas et al. [115] investigated elevated PD‐L1 and Vim expression on CTCs in metastatic versus early‐stage patients, both of which are associated with reduced PFS and OS. Similarly, Todenhöfer et al. [116] described vim^+^ CTC enrichment in advanced disease, whereas Liu et al. [117] demonstrated epithelial‐mesenchymal plasticity in CTCs as a determinant of lung metastatic tropism.

Although CTCs have been shown to act as biomarkers of poor prognosis in BC patients, sufficient evidence to supersede established prognostic determinants such as primary tumor size (T), nodal involvement (N), or genetic analysis is currently lacking [22, 118] (Figure 1).

### 4.4 Exosomes in BC: From CTC Dynamics to Nanomedicine Innovations

Exosomes are subtypes of nanosized (30 to 150 nm) vesicles released from most cell types into biological fluids and are characterized by a lipid bilayer membrane encapsulating functional biomolecules such as nucleic acids (DNA, RNA), proteins, and lipids [10, 119]. Notably, cancer cells, including CTCs, exhibit greater exosome secretion than normal cells do, with tumor‐derived exosomes (TDEs) serving as molecular snapshots of parental cell states [12]. These TDEs not only reflect tumor dynamics but also hold promise as non‐invasive biomarkers for real‐time therapeutic monitoring.

Emerging evidence underscores the pivotal role of exosomes in BC progression through multiple mechanisms: TDEs facilitate tumor invasion and metastasis by remodeling the tumor microenvironment (TME) [5, 12, 120]. They transport proangiogenic mediators (e.g., VEGF and MMPs) to endothelial cells, driving neovascularization, which is critical for metastatic dissemination [121, 122]. TDEs mediate intercellular communication between drug‐resistant and drug‐sensitive cancer cells via the transfer of proteins, miRNAs (e.g., miR‐23b), and other cargo. This horizontal transfer induces multidrug resistance (MDR) by promoting dormancy and evading chemotherapeutic cytotoxicity [122, 123]. Leveraging their biocompatibility, membrane permeability, and ability to bypass P‐glycoprotein‐mediated drug resistance, exosomes outperform synthetic nanocarriers as targeted chemotherapeutic delivery systems [122, 124]. TDEs express tumor‐specific antigens capable of eliciting antitumor immune responses, positioning them as ideal vectors for next‐generation nanovaccines against BC [122, 125].

Collectively, exosomes released by tumor cells serve as carriers of diverse biomarkers and have been implicated in pivotal mechanisms underlying the pathophysiology of BC [120].

### 4.5 NcRNAs in BC CTCs: Biomarkers and Clinical Implications

NcRNAs are functional RNA molecules transcribed from DNA but not translated into proteins. On the basis of their nucleotide length, ncRNAs are categorized into two major types: long noncoding RNAs (lncRNAs; > 200 nucleotides) and small noncoding RNAs (sncRNAs; < 200 nucleotides) [126]. LncRNAs play a regulatory role in protein and miRNA function and expression levels [127]. SncRNAs can be classified into diverse subtypes, including miRNAs, small nucleolar RNAs (snoRNAs), and small nuclear RNAs (snRNAs), with miRNAs being the most extensively studied in oncology [126, 128]. Notably, miRNAs are actively secreted by CTCs and tumor cells into the extracellular milieu as circulating miRNAs [11, 129, 130, 131]. CTC‐derived miRNAs serve as ideal biomarkers for real‐time tumor profiling, as dysregulation of specific miRNAs is correlated with disease progression, enabling dynamic risk stratification and therapeutic assessment [132, 133].

Tumor‐associated miRNA profiling has demonstrated clinical utility in identifying high‐risk populations for BC development [129]. Elevated miRNA levels are observed in CTC‐positive patients, reflecting their role in oncogenesis. miRNAs can promote or inhibit tumor development by up‐ or downregulating the expression of oncogenes and tumor suppressor genes (TSGs). For example, compared with conventional biomarkers (e.g., CA153 and CEA), circulating miR‐21 has superior diagnostic sensitivity for early‐stage BC [133, 134], primarily through the targeting of oncogenic pathways [129]. Furthermore, miR‐155, miR‐19a, and other miRNAs known to suppress TSGs are significantly upregulated in ductal carcinoma in situ (DCIS) and invasive ductal carcinoma (IDC) compared with normal tissue and atypical ductal hyperplasia (ADH) in serum samples, suggesting their potential in differentiating BC stages [135]. In addition to single‐miRNA assays, combinatorial miRNA panels enhance diagnostic precision. A study utilizing a 9‐miRNA panel demonstrated higher overall expression in BC at stages I, II, and III than in stage IV, indicating stage‐specific molecular dynamics [136].

miRNAs also hold prognostic significance. Elevated miR‐21 expression is correlated with reduced OS and increased recurrence risk in BC [137], whereas miR‐10b—a driver of cell migration and invasion—is linked to metastatic progression and poor prognosis [138]. Similarly, miR‐155, which modulates immune evasion and inflammatory pathways, is associated with advanced lymph node metastasis and unfavorable outcomes [132, 139].

In therapeutic monitoring, dynamic miRNA quantification provides actionable insights. Postoperative and postchemoradiation decreases in miR‐21 and miR‐155 levels reflect treatment efficacy, positioning miRNAs as biomarkers for response assessment [132, 140]. In HER2‐positive BC, neoadjuvant trastuzumab therapy reduces miR‐21 expression, underscoring its utility in monitoring HER2‐targeted regimens [141].

Despite these advances, CTC‐based miRNA analysis faces methodological challenges, including low specificity, technical limitations in enrichment, and heterogeneity in miRNA cargo [142]. To address this, multi‐miRNA panels—rather than single‐miRNA assays—are advocated to improve diagnostic accuracy, treatment monitoring, and long‐term management in BC [143, 144]. Integrating miRNA profiling with other biomarkers may further enhance clinical utility [145, 146].

## 5 Challenges and Future Perspectives

To fully harness the clinical potential of CTCs and CTECs in advancing multidisciplinary cancer management—encompassing early disease stratification, therapeutic optimization, real‐time efficacy evaluation, therapy resistance surveillance, minimal residual disease (MRD) detection, and posttreatment recurrence monitoring—integration of multiparametric analyses (including genomic, transcriptomic, proteomic, and morphological profiling) is imperative [147, 148]. Despite accumulating evidence supporting their prognostic and predictive utility, there are still numerous obstacles and room for progress. Given tumors' intrinsic heterogeneity, CTCs—as biomarkers reflecting in vivo tumor characteristics—inevitably exhibit heterogeneity [149]. Whereas this poses technical challenges in detection, it partially compensates for the limited information provided by single‐site biopsies [150]. Heterogeneity in CTC/CTEC, coupled with the absence of standardized protocols for capturing all subtypes (e.g., EMT‐induced or necrotic CTCs), restricts their application [9, 151]. We propose the development of unified and standardized CTC/CTEC detection methods and analytical methodologies to increase the clinical utility, accuracy, and cost‐effectiveness of these methods. Additionally, CTC/CTEC cannot guide diagnosis or treatment as a clinical standard alone but can only be used as an auxiliary tool. Current screening tools must meet criteria such as speed, convenience, affordability, and non‐invasiveness [152]. While CTC detection is non‐invasive and addresses limitations of conventional methods, its higher cost and longer turnaround time (3–5 days versus immediate imaging results) currently limit its utility as a routine screening tool [29, 63]. More evidence is needed to support their independent role. Consequently, there is still a gap between clinical research and routine clinical adoption.

Emerging evidence highlights that the quantity of CTCs varies in response to circadian rhythms and hormone levels, peaking at rest [153], necessitating chronobiology‐informed sampling strategies to maximize detection sensitivity. Currently, the indicators of efficacy are the change in CTC count and the proportion of necrotic cells, but there are no standardized criteria. In the future, a precise standard value or range for the proportion of necrotic cells will need to be formulated to clarify whether and to what extent the treatment is effective. Furthermore, trophoblast cell surface antigen‐2 (Trop‐2) has emerged as a therapeutically exploitable target, with Trop‐2‐directed antibody–drug conjugates (ADCs) demonstrating efficacy across multiple malignancies, such as prostate, lung, and breast cancer [154, 155, 156]. Additionally, they can benefit people who have become resistant to medications. The expression of Trop‐2 on CTCs could be the subject of additional clinical trial research and could be the focus of upcoming medication development.

CTECs, which are characterized by their hybrid epithelial–endothelial–mesenchymal attributes and function as both vascular mediators and malignant propagators, are thought to play a significant role in the development, spread, and metastasis of tumors [103, 110]. In regard to treatment resistance and disease progression, eliminating CTECs in cancer patients may be another way to prevent the spread of cancer effectively.

CTC detection technologies have evolved from forward‐enrichment approaches to emerging reverse‐enrichment methods. The former demonstrates high specificity but has limited sensitivity and a narrow clinical applicability population [26]. Nonetheless, its clinical value has been validated through Phase III trials in lung/breast cancer and regulatory approvals [23, 157]. Although the CTC reverse enrichment method reduces specificity, its sensitivity is significantly improved, making it suitable for a wider population, including early‐stage and follow‐up patients [62]. However, strict design, large sample size, and randomized controlled clinical research results are required to be accepted by regulatory authorities and clinical practice. In the future, our focus should be on improving technology, reducing false positives, establishing population‐specific cutoff values, and conducting Phase III studies to confirm clinical efficacy.

## 6 Conclusion

Recent technological breakthroughs in CTC detection, particularly SE‐iFISH, have significantly improved the sensitivity and specificity of CTC identification while enabling comprehensive molecular characterization. These innovations have prompted a resurgence in CTC research, particularly in understanding their role in tumor biology and metastatic progression. Clinically, adopting such technologies has now expanded the utility of CTC, offering real‐time monitoring of tumor dynamics, including genetic alterations, marker expression, and therapeutic responses.

This review systematically delineates the clinical significance of CTCs in BC. By synthesizing current evidence, we emphasize the potential of CTCs and their derivatives to serve as dynamic biomarkers for tumor burden, metastasis, and treatment resistance. Notably, advanced codetection strategies for aneuploid CTCs and CTECs enhance patient management by enabling early diagnosis, risk stratification, and personalized therapeutic interventions. Our analysis underscores the imperative to translate experimental discoveries into clinical workflows, ultimately improving outcomes in BC and broader malignancies.

## Author Contributions

The corresponding author takes full responsibility that all authors on this publication have met the following required criteria of eligibility for authorship: (a) significant contributions to the design of article concepts and frameworks; (b) drafting or revising the article for intellectual content; (c) final approval of the published article; and (d) agreement to be accountable for all aspects of the article thus ensuring that questions related to the accuracy or integrity of any part of the article are appropriately investigated and resolved. Nobody who qualifies for authorship has been omitted from the list.

## Disclosure

The work has never been published nor is under consideration elsewhere, that all authors agree with its content, and agree to transfer the copyright to FESEO if accepted for publication.

## Conflicts of Interest

The authors declare no conflicts of interest.

## Data Availability

Data sharing not applicable to this article as no datasets were generated or analysed during the current study.
